# Depression Treatment among Adults with Multiple Sclerosis and Depression in Ambulatory Care Settings in the United States

**DOI:** 10.1155/2017/3175358

**Published:** 2017-04-27

**Authors:** Sandipan Bhattacharjee, Lisa Goldstone, Queeny Ip, Terri Warholak

**Affiliations:** Department of Pharmacy Practice and Science, College of Pharmacy, The University of Arizona, Tucson, AZ, USA

## Abstract

*Background*. There is little information regarding depression treatment patterns among adults with MS and depression in ambulatory settings at national level in the United States (US).* Objectives*. The objectives of this study were to identify patterns and predictors of depression treatment in ambulatory settings in US among adults with MS and depression.* Methods*. A cross-sectional study was conducted by pooling multiple years (2005–2011) of National Ambulatory Medical Care Survey and the outpatient department of the National Hospital Ambulatory Medical Care Survey data. The final study sample was comprised of ambulatory visits among adults with MS and depression. Dependent variable of this study was pharmacological treatment for depression with or without psychotherapy. Predictors of depression treatment were determined by conducting multivariable logistic regression.* Results*. Out of all ambulatory visits involving MS diagnosis, 20.59% also involved a depression diagnosis. Depression treatment was observed in 57.25% of the study population. Fluoxetine was the most prescribed individual antidepressant. Age and total number of chronic diseases were significant predictors of depression treatment.* Conclusion*. Approximately six out of ten ambulatory visits involving MS and depression recorded some form of depression treatment. Future longitudinal studies should examine health outcomes associated with depression treatment in this population.

## 1. Introduction

Multiple Sclerosis (MS) is a disabling central nervous system disease, which is estimated to affect nearly 400,000 individuals in the United States (US) and around 2.3 million individuals worldwide [1]. Individuals with MS suffer from a wide array of physical and psychiatric comorbidities, with depression being one of the most common psychiatric comorbidities [2]. Existing studies suggest that the prevalence and lifetime incidence of depression among individuals with MS are approximately twofold and threefold higher, respectively, compared to those without MS [3, 4]. The presence of depression is further complicated by chronic pain among nearly 50% of individuals with MS [5]. Antidepressants and psychotherapy have been observed to be effective in the treatment of depressive symptoms among individuals with MS [4, 6]. However, according to a review conducted by Skokou et al. (2012), depression in MS patients is highly underdiagnosed and when these patients are identified, they remain undertreated for depressive symptoms [7].

Despite the importance of treatment of depression among individuals with MS, there have been very few studies examining depression treatment in this population. The majority of the studies examining the use of depression treatment have been conducted in international settings [8–10], while only two were conducted in the US [11, 12]. A cross-sectional study conducted by Cetin et al. (2007) in Eastern Washington State examined the prevalence of depressive symptoms and the frequency of antidepressant use among members of the Spokane chapter of the National Multiple Sclerosis Society (NMSS) using a mail survey among those with MS [12]. This study reported that 40% of those with clinically significant depressive symptoms were taking antidepressants, leaving nearly 60% untreated. Another study conducted by Mohr et al. (2006), examined the antidepressant use among individuals with MS who were treated by neurologists at Kaiser Permanente of Northern California [11]. This study found that around 65% of MS patients did not receive antidepressant treatment and less than one-third received threshold antidepressant dose or over. Although these findings are informative, the geographic and time limitations of these studies conducted in US do not permit the examination of the prescribing patterns, and inferences at national level. To the best of our knowledge, no study has examined the prescribing patterns and predictors of depression treatment among adults with MS and depression at national level in the US. National level information could provide insights into possible opportunities to improve treatment outcomes in MS patients in the US. Therefore, the goal of this study was to identify patterns and predictors of depression treatment in ambulatory settings adults with MS and depression in the US.

## 2. Methods

### 2.1. Study Design

We adopted a cross-sectional study design by pooling data from National Ambulatory Medical Care Survey (NAMCS) and the outpatient department (OPD) of the National Hospital Ambulatory Medical Care Survey (NHAMCS) (2005–2011). This study was deemed to be Not Human Subjects Research by the Institutional Review Board of The University of Arizona.

### 2.2. Data Source

NAMCS and NHAMCS are nationally representative sample of the health care services delivered to individuals in the US ambulatory settings. NAMCS and NHAMCS are annual ongoing surveys conducted by the National Center for Health Statistics (NCHS) of the Centers for Disease Control and Prevention (CDC) [13]. National estimates describing the utilization of ambulatory medical care services in the US are produced by using the weights provided in these data [14].

Both NAMCS and NHAMCS data are collected by administering cross-sectional surveys, to capture information on the physician-patient encounter or visit, which serves as the basic sampling unit. A direct, personal exchange between a physician, or a staff member working under a physician's direction, for the purpose of seeking care and providing health services, is defined as a visit [15]. NAMCS uses a multistage probability design involving samples of primary sampling units (counties, groups of counties, county equivalents, or towns and townships), practices of physicians inside primary sampling units, and patient visits within each practice. The first probability sample is drawn from these primary sampling units. The next probability sample is drawn from the practicing physicians from each of these primary sampling units. Lastly, the patient visits within the annual practices of sample physicians are selected in a two-step process. Firstly, the entire physician sample is divided into 52 random subsamples of nearly equal size, and each of these subsamples is randomly assigned to the 1 week of the 52 weeks of the survey year. Secondly, a systematic random sample of visits is selected by the physicians during the assigned week. Extensive information regarding patient characteristics, physician characteristics, physician's diagnoses, prescribed pharmacotherapy, and the delivery of therapeutic services was requested in the NAMCS data collection form [also known as the Patient Record Form (PRF)]. International Classification of Diseases, 9th Revision, Clinical Modification (ICD-9-CM) code was used to report diagnosis and a maximum of three diagnoses could be reported. Up to eight prescription medications were recorded for each visit [14].

NHAMCS collects data from nonfederal and noninstitutional hospitals across the US. A multistage probability sample survey is used to collect data from a representative sample of visits to outpatient and emergency departments [15]. The survey design involves selection of probability samples of PSUs, hospitals from each PSU, some or all outpatient and emergency departments from hospitals, and patient visits within these departments. The final stage of sampling in the NHAMCS is similar to that of the NAMCS. Only the OPD portion of the NHAMCS was used for this study because the medical care provided in these settings is similar to the care provided in office-based settings. The clinical nature of the OPD visits was collected using a PRF similar to the one used in the NAMCS.

### 2.3. Study Population

The study sample was identified from ambulatory visits among adults (age ≥ 18 years) with MS and depression. Ambulatory visits with MS were identified by using ICD-9-CM of 340.xx [16], while visits with depression were identified by ICD-9-CM codes of 296.2–296.36, 300.4, or 311, or if the answer to the question “Regardless of the diagnoses written…does the patient now have: depression?” was “yes” [17]. The latter item was available from 2005 onward to supplement chronic disease related ambulatory visits and to overcome the issue of underestimation of chronic conditions. The robustness of this item has been described in detail elsewhere [18].

### 2.4. Dependent Variable

Dependent variable for our study was depression treatment, which consisted of antidepressant use with or without psychotherapy. Antidepressant use was ascertained by using the Multum Lexicon Code as well as Generic Drug Code. Antidepressants were categorized into the following subclasses as per the 2016 American Hospital Formulary Service (AHFS) classification system (The Appendix): (i) Selective serotonin reuptake inhibitors (SSRIs); (ii) serotonin–norepinephrine reuptake inhibitors (SNRIs); (iii) tricyclic antidepressants (TCAs); (iv) monoamine oxidase inhibitor (MAO inhibitors); (v) serotonin modulators; and (vi) miscellaneous antidepressants [19]. Psychotherapy use was identified using the variable “PSYCHOTH” (yes/no) from NAMCS and NHAMCS data.

### 2.5. Independent Variables

Based on the Anderson Behavioral Model (ABM), we categorized the independent variables into (i) predisposing; (ii) enabling; and (iii) need factors [20]. Predisposing factors consisted of age (18–39 years and ≥40 years); gender (male/female); race/ethnicity (White Only Non-Hispanic and others); geographical region (Northeast, Midwest, West, and South); and metro status (metro, nonmetro). Enabling factors comprised of insurance (government insurance such as Medicaid/Medicare and others); and physician/clinic specialty (general and family practice and others). The need factors constituted of reason for visit (chronic problem or routine; others); whether new medications were prescribed during the visit (yes/no); use of disease modifying drugs (Interferon Beta1A, Glatiramer, Daclizumab, Fingolimod, Mitoxantrone, and Natalizumab); and chronic diseases [arthritis, asthma, cancer, congestive heart failure (CHF), chronic obstructive pulmonary disease (COPD), diabetes, hyperlipidemia, hypertension, ischemic heart disease (IHD), and anxiety]. These chronic diseases were coded yes/no using the question: “Regardless of the diagnoses written…does the patient now have:…?”

### 2.6. Statistical Analysis

National estimates were obtained by adjusting for the complex survey design of NAMCS-NHAMCS using SAS version 9.4 (SAS institute Inc., Cary, NC, USA). Descriptive statistics were reported in terms of weighted frequencies and percentages for the number of visits at national level. Predictors of depression treatment were ascertained by conducting multivariate logistic regression (adjusted for predisposing, enabling, and need factors) with any depression treatment versus no treatment as the dependent variable.

## 3. Results

According to the study findings, in 2005–2011 approximately 10.22 million visits involved a diagnosis of MS, representing nearly 0.17% of all ambulatory visits at national level among US adults. Among these 10.22 million visits, depression was recorded in 2.1 million visits (20.59%, 95% CI, 14.85%–26.32%), thus forming our final study population.

Table 1 provides the demographic and clinical characteristics of adults with MS and depression in terms of predisposing, enabling, and need factors. This table reports the weighted frequencies of visits in millions (national level) as well as their corresponding weighted percentages. Majority of the visits involving our study population were aged 40 years or older (84.55%), were female (76.2%), were Non-Hispanic Whites (89%), resided in metro regions (88.6%), and had nongovernment insurances (65.08%). Approximately 70% of the visits involving our study population were from physician/clinic specialties other than general and family practice and were in the Midwest (35.82%), followed by the South (29.62%), the Northeast (19.35%), and the West (15.21%). Nearly half (48.68%) of the visits involving our study population were routine or chronic problem related and an overwhelming majority were established patients (96.21%). In approximately one out of five visits in our study population a prescription for a new medication was recorded. Disease modifying drugs were prescribed in 27.11% of the visits involving our study population; some of the most common diagnoses in our study population were hypertension (34.47%), hyperlipidemia (19.03%), arthritis (17.53%), COPD (8.7%), and diabetes (8.14%). Mean total number of medications used and total chronic conditions present in our study population were 4.42 (SE 0.25) and 2.14 (SE 0.09), respectively (data not presented in tabular form).

Depression treatment patterns in our study population are presented in Table 2. The overall depression treatment (antidepressant with or without psychotherapy) was observed in 57.25% (95% CI, 46.29%–68.19%) of our study population. Antidepressants were prescribed in 55.54% (95% CI, 44.36%–66.73%), whereas psychotherapy was used in 4.48% (95% CI, 1.82%–7.14%) of the visits in our study population. Among subclasses of antidepressants, SSRIs were the most prescribed (55.67%, 95% CI 38.14%–73.27%) followed by SNRIs (35.04%, 95% CI 16.98%–53.11%), miscellaneous antidepressants (6.97%, 95% CI 1.47%–12.48%), serotonin modulators (5.08%, 95% CI 0.52%–9.65%), and TCAs (3.32%, 95% CI 0.0%–7.44%) in our study population. In terms of individual antidepressants, some of the most commonly prescribed ones included fluoxetine (20.05%), duloxetine (17.76%), escitalopram (13.16%), desvenlafaxine (10.04%), sertraline (8.99%), paroxetine (8.18%), venlafaxine (7.85%), citalopram (5.32%), and trazodone (5.08%) in our study population. Psychotherapy was recorded in 4.48% (95% CI, 1.82%–7.14%) of the study sample representing around 0.094 million ambulatory visits.

Table 3 exhibits the findings from multivariate logistic regression analyses to examine the predictors of depression treatment in our study population. Individuals who were 40 years or older were 81% less likely [Adjusted Odds Ratio (AOR): 0.189, 95% CI 0.036–0.997] to receive depression treatment compared those who were in the age group of 18–39 years. With the increase in each chronic condition, the likelihood of receiving depression treatment decreased by 44% (AOR: 0.554, 95% CI 0.335–0.917).

## 4. Discussion

MS is a chronic condition that requires adherence to prescribed treatment regimens to avoid relapses and hospitalizations, and untreated depression is associated with nonadherence for many disease states including MS [21]. Additionally, depressive symptoms may place these patients at greater risk for suicide [22]. Hence, understanding depression treatment patterns among individuals with MS and depression is critical and our study adds value to the existing literature by providing this information at national level. The visit-level prevalence of depression among individuals with MS identified in the current study (20.59%) is consistent with both a recent systematic review and meta-analysis, which estimated the prevalence of depression among individuals with MS to be around 24% [2] and the point prevalence of major depressive disorder in approximately 26% of individuals with MS observed in a study among patients recruited by Northern California Kaiser Permanente Medical Care Group [11]. However, our estimate is lower than the depression prevalence estimate of 51% among a sample of individuals with MS who were members of the Spokane (Washington) chapter of the National Multiple Sclerosis Society (NMSS) [12] in which presence of depression was self-reported by using the Center for Epidemiologic Studies Depression Scale (CES-D) [12]. Variations in the prevalence rates of depression observed in different studies can be attributed to the types of depression assessment measures used and possible underdiagnosis of depression as well as stigma associated with reporting mental disorder.

Receipt of some form of depression treatment was reported by around 6 out of 10 ambulatory visits in our study. Our estimate of antidepressant use (55.54%) among individuals with MS and depression is higher compared to 41% estimated by the NMSS Spokane chapter study [12] and 34% by Northern California Kaiser Permanente Medical Care Group study. It is challenging to elucidate the reason for these differences in antidepressant use from the current evidence. However, it is possible that the higher antidepressant use may be due to these medications being used for indications other than depression. Duloxetine, which accounted for nearly 18% of antidepressant therapies used in this population, is used to treat both neuropathic and chronic musculoskeletal pain whereas trazodone is commonly prescribed for insomnia especially among patient populations who may be prone to abuse or misuse controlled sedatives and hypnotics. One can speculate that there may be regional differences in antidepressant use among individuals with MS and depression across US although regional variation was not observed in our study. Further the researches is warranted to understand the underlying reasons for this difference. However, none of the existing studies examined the proportion of individuals with MS and depression treated with psychotherapy. Psychotherapy, particularly Cognitive Behavioral Therapy (CBT), is recommended for individuals with MS and depression according to the Goldman Consensus group [4]. Efficacy of CBT has been evaluated in existing randomized controlled trials among individuals with MS and depression; response rates were similar or higher than antidepressant treatment [23, 24]. Our study found a very low utilization of psychotherapy (4.48% of the study sample). This treatment gap presents an opportunity for healthcare providers to consider psychotherapy as a viable treatment option for individuals with MS.

It was not surprising that SSRIs were identified as the most prescribed subclass of antidepressants in our study as SSRIs along with other newer antidepressants usually have better adverse events profile among antidepressants and hence are expected to be frequently prescribed for patients with depression who are already coping with MS symptoms [25]. Among individual antidepressants, fluoxetine was the most prescribed in our study population. This finding is consistent with the finding from the NMSS Spokane chapter study [12]. Preference of fluoxetine among individuals with MS is not unexpected as fluoxetine has been observed in studies to reverse axonal dysfunction in this population [26]. A double-blind, randomized, placebo controlled trial found that fluoxetine was usually well tolerated among individuals with MS and no effect was observed on disability progression [27]. This trial was however underpowered and future trials with adequate sample size is required to make appropriate inferences. Moreover, animal models have provided increasing evidence of the possible neuroprotective and neurodegenerative actions of fluoxetine [28]. Duloxetine was the second most prescribed antidepressants observed in our study, which is not unexpected because an open-label study demonstrated its safety, tolerance, and efficacy in reducing depression among individuals with MS [29]. Existing studies have also demonstrated the benefits of using other commonly prescribed antidepressants such as escitalopram [30], sertraline [31], and paroxetine [32] which were observed in our study to a lesser degree.

Our study findings suggested that older individuals (age ≥ 40 years) were significantly less likely to receive any form of depression treatment compared to younger individuals (age 18–39 years). A study by Conner et al. (2010) among older adults with depression concluded that due to a high level of public stigma associated with depression diagnosis, older adults were unlikely to be engaged in or to seek mental health care [33]. Findings from the Conner et al. (2010) [33] study reflect the findings of our study, where older adults were less likely to receive depression treatment compared to young adults with MS and depression. However, further research is needed to understand the underlying reasons for this age-related difference in depression treatment among the study population. Our study findings also suggested that increase in the number of chronic diseases was associated with decreased likelihood of receiving depression treatment. Some of the reasons which may explain this finding are competing demands associated with other disease conditions [34]; increased risk of adverse events (such as falls, injurious falls, and greater number of falls) associated with polypharmacy including antidepressants [35]; and possibility of drug interactions [36].

Several limitations should be kept in mind while interpreting the findings from this study. The final unweighted study sample (*N* = 220) was small and hence may lead to statistical underpower. NAMCS and NHAMCS databases provide up to 3 diagnoses per visit, which can potentially lead to underestimation of disease conditions. However, the use of antidepressants for other conditions such as sedation, pain, sleep, and appetite stimulation is possible which may lead to overestimation of patients who are prescribed antidepressants for depression. Information concerning the duration and severity of MS and depression; dosage information; activities of daily living, instrumental activities of daily living, and functional status was not available. Patient and physician preferences, which are important factors for treatment selection, were also not available in the datasets. Moreover, to achieve appropriate Relative Standard Error, several variable categories (such as region, insurance) had to be combined to achieve reliable estimates. Furthermore, types of psychotherapy were not available in NAMCS and NHAMCS datasets. Other usual limitations associated with secondary data such as the possibility for reporting errors, coding errors, and interviewer effects should also be considered. Finally, due to the cross-sectional study design, causal inferences cannot be made.

## 5. Conclusion

Data from this cross-sectional, retrospective database study indicates that concurrent diagnoses of depression and MS are not uncommon. About half of those with both diagnoses received pharmacotherapy (most often SSRIs, specifically fluoxetine). Younger age and lower number of chronic diseases were associated with the receipt of depression treatment among those with MS and depression. Future longitudinal studies should examine clinical and economic outcomes associated with depression treatment in those with MS.

## Figures and Tables

**Table 1:** Demographic and clinical characteristics of adults with MS and depression (NAMCS-NHAMCS 2005–2011).

Characteristics	Unweighted freq	Wt. freq (millions)	Wt.%
Predisposing factors			

*Age*			
18–39	50	0.325	15.45
≥40	170	1.778	84.55
*Gender*			
Male	51	0.501	23.80
Female	169	1.604	76.20
*Race/ethnicity*			
White Only, NH	172	1.873	89.00
Others	48	0.232	11.00
*Geographic region*			
West	37	0.320	15.21
Northeast	56	0.407	19.35
Midwest	63	0.754	35.82
South	64	0.623	29.62
*Metro status*			
Metro	204	1.864	88.60
Nonmetro	16	0.240	11.41

Enabling factors			

*Insurance*			
Govt. insurance	97	0.735	34.93
Others	123	1.369	65.08
*Physician/clinic specialty*			
Gen & fam prac	42	0.639	30.39
Others	178	1.465	69.61

Need factors			

*Reason visit*			
Chron prob/routine	143	1.024	48.68
Others	77	1.080	51.32
*New prescription during visit*			
Yes	65	0.552	26.22
No	155	1.552	73.78
*Disease modifying drugs*			
Yes	81	0.570	27.11
No	139	1.534	72.89
*Chronic diseases*			
ARTHRITIS	20	0.369	17.53
ASTHMA	9	0.037	1.78
CANCER	7	0.069	3.27
CHF	2	0.031	1.48
COPD	5	0.183	8.70
DIABETES	17	0.171	8.14
HYPLIPID	33	0.400	19.03
HTN	49	0.725	34.47
IHD	3	0.045	2.11
ANXIETY	3	0.039	1.85

*Note*. Based on 220 (nationally representative weighted *N* = 2.104 million) ambulatory visits of adults (age ≥ 18 years) with Multiple Sclerosis and depression using NAMCS and NHAMCS 2005–2011 data.

MS: Multiple Sclerosis; NAMCS: National Ambulatory Medical Care Survey; NHAMCS: National Hospital Ambulatory Medical Care Survey; Wt.: weighted; freq: frequency; NH: Non-Hispanic; Gen & fam prac: general and family practice; Govt: government; Chron prob: chronic problem; Estab: established; CHF: congestive heart failure; COPD: chronic obstructive pulmonary disease; HYPLIPID: hyperlipidemia; HTN: hypertension; IHD: ischemic heart disease.

**Table 2:** Depression treatment pattern among adults with MS and depression (NAMCS-NHAMCS 2005–2011).

	Unweighted freq	Wt. freq (millions)	Wt.%
*Overall depression treatment*	*133*	*1.205*	*57.25*
*Overall psychotherapy use*	18	*0.094*	*4.48*
*Overall antidepressant use*	126	*1.169*	*55.54*

Antidepressant class and individual antidepressant use			

*SNRI*	32	*0.417*	*35.04*
Desvenlafaxine	2	0.117	10.04
Duloxetine	13	0.208	17.76
Venlafaxine	18	0.092	7.85
*SSRI*	78	*0.651*	*55.67*
Citalopram	9	0.062	5.32
Escitalopram	19	0.154	13.16
Fluoxetine	20	0.234	20.05
Paroxetine	5	0.096	8.18
Sertraline	25	0.105	8.99
*TCAs*	11	*0.07*	*3.32*
Amitriptyline	4	0.015	1.25
Desipramine	1	0.003	0.29
Doxepin	2	0.043	3.63
Nortriptyline	4	0.009	0.80
*Serotonin Modulators*	12	*0.107*	*5.08*
Trazodone	12	0.107	5.08
*Miscellaneous*	15	*0.147*	*6.97*
Bupropion	11	0.069	3.25
Mirtazapine	4	0.078	3.72

*Note*. Based on 220 (nationally representative weighted *N* = 2.104 million) ambulatory visits of adults (age ≥ 18 years) with Multiple Sclerosis and depression using NAMCS and NHAMCS 2005–2011 data.

MS: Multiple Sclerosis; Wt.: weighted; freq: frequency; NAMCS: National Ambulatory Medical Care Survey; NHAMCS: National Hospital Ambulatory Medical Care Survey.

Did not observe use of the following antidepressants: Amoxapine, Clomipramine, Imipramine, Maprotiline, Protriptyline, Trimipramine, Vilazodone, Vortioxetine, and Nefazodone.

**Table 3:** Predictors of depression treatment among adults with MS and depression (NAMCS-NHAMCS 2005–2011).

Characteristics	AOR	95% CI	Sig
Predisposing factors			

*Age* ^*∗*^			
18–39	Ref		
≥40	0.189	[0.036, 0.997]	0.0496
*Gender*			
Female	Ref		
Male	0.970	[0.303, 3.104]	0.9591
*Race/ethnicity*			
White Only, NH	0.848	[0.214, 3.360]	0.8144
Other	Ref		
*Georegion*			
South	1.187	[0.475, 2.962]	0.7138
Other	Ref		
*Metro*			
Metro	Ref		
Nonmetro	0.929	[0.172, 5.034]	0.9321

Enabling Factors			

*Physpec/clinical specialty*			
Gen & fam prac	Ref		
Others	0.884	[0.216, 3.617]	0.8639
*Insurance*			
Govt. insurance	Ref		
Others	3.379	[0.924, 12.354]	0.0656

Need Factors			

*Reason visit*			
Chronic prob, rout	Ref		
Others	0.734	[0.256, 2.107]	0.5658
*New prescription*			
No new meds	Ref		
≥1 new med	0.356	[0.118, 1.070]	0.0659
*Disease modifying drugs*			
Yes	1.115	[0.314, 3.955]	0.8662
No			
*NUMMED*	1.352	[0.987, 1.852]	0.0604
*TOTCHRON* ^*∗*^	0.554	[0.335, 0.917]	0.0216

*Note*. Based on 220 (nationally representative weighted *N* = 2.104 million) ambulatory visits of adults (age ≥ 18 years) with Multiple Sclerosis and depression using NAMCS and NHAMCS 2005–2011 data.

MS: Multiple Sclerosis; NAMCS: National Ambulatory Medical Care Survey; NHAMCS: National Hospital Ambulatory Medical Care Survey; Ref: reference group; AOR: Adjusted Odds Ratio; CI: confidence interval; Sig: significance; NH: Non-Hispanic; Physpec: Physician Specialty; Gen & fam prac: general and family practice; Chron prob: chronic problem; NUMMED: total number of medications; TOTCHRON: total number of chronic conditions.

^*∗*^ *Statistically significant at p* < 0.0*5.*

**Table 4:** List of antidepressant drug categories.

Drug classes	Individual drugs
Antidepressants	*MAO inhibitors*:
	Phenelzine (Nardil)
	Tranylcypromine (Parnate)
	*SNRI*:
	Desvenlafaxine (Khedezla; Pristiq)
	Duloxetine (Cymbalta; Irenka)
	Levomilnacipran (Fetzima; Fetzima Titration)
	Venlafaxine (Effexor XR)
	*SSRI*:
	Citalopram (CeleXA)
	Escitalopram (Lexapro)
	Fluoxetine (PROzac; PROzac Weekly; Sarafem)
	Fluvoxamine (Luvox CR)
	Paroxetine (Brisdelle; Paxil; Paxil CR; Pexeva)
	Sertraline (Zoloft)
	*Serotonin modulators*:
	Nefazodone (Nefazodone Hydrochloride)
	Trazodone (Oleptro)
	Vilazodone (Viibryd; Viibryd Starter Pack)
	Vortioxetine (Trintellix, Brintellix)
	*Tricyclics and other norepinephrine-reuptake inhibitors*:
	Amitriptyline (Elavil)
	Amoxapine (Amoxapine)
	Clomipramine (Anafranil)
	Desipramine (Norpramin)
	Doxepin Hydrochloride (systemic: Silenor Topical: Prudoxin; Zonalon)
	Imipramine (Tofranil; Tofranil-pm)
	Maprotiline (Maprotiline Hydrochloride)
	Nortriptyline (Pamelor)
	Protriptyline (Vivactil)
	Trimipramine (Surmontil)
	*Miscellaneous*:
	Bupropion (Aplenzin; Forfivo XL; Wellbutrin SR; Wellbutrin XL;
	Wellbutrin; Zyban)
	Mirtazapine (Remeron; Remeron SolTab)
	Atomoxetine Hydrochloride (Strattera)

*Source. AHFS 2016 available at*: https://online.statref.com/Document.aspx?

## Appendix

See Table 4.

## References

[1] National Multiple Sclerosis Society (NMSS) http://www.nationalmssociety.org/About-the-Society/MS-Prevalence.

[2] Marrie R. A., Cohen J., Stuve O. (2015). A systematic review of the incidence and prevalence of comorbidity in multiple sclerosis: overview. *Multiple Sclerosis Journal*.

[3] Kessler R. C., Berglund P., Demler O. (2003). The epidemiology of major depressive disorder: results from the National Comorbidity Survey Replication (NCS-R). *Journal of the American Medical Association*.

[4] Goldman Consensus Group (2005). The Goldman Consensus statement on depression in multiple sclerosis. *Multiple Sclerosis (Houndmills, Basingstoke, England)*.

[5] O'Connor A. B., Schwid S. R., Herrmann D. N., Markman J. D., Dworkin R. H. (2008). Pain associated with multiple sclerosis: systematic review and proposed classification. *Pain*.

[6] Schumann R., Adamaszek M., Sommer N., Kirkby K. C. (2012). Stress, depression and antidepressant treatment options in patients suffering from multiple sclerosis. *Current Pharmaceutical Design*.

[7] Skokou M., Soubasi E., Gourzis P. (2012). Depression in multiple sclerosis: a review of assessment and treatment approaches in adult and pediatric populations. *ISRN Neurology*.

[8] Brenner P., Alexanderson K., Bjorkenstam C. (2014). Psychiatric diagnoses, medication and risk for disability pension in multiple sclerosis patients; a population-based register study. *PLoS ONE*.

[9] Seyed Saadat S. M., Hosseininezhad M., Bakhshayesh B., Seyed Saadat S. N., Nabizadeh S. P. (2014). Prevalence and predictors of depression in Iranian patients with multiple sclerosis: a population-based study. *Neurological Sciences*.

[10] Sollom A., Kneebone II. (2007). Treatment of depression in people who have multiple sclerosis. *Multiple Sclerosis*.

[11] Mohr D. C., Hart S. L., Fonareva I., Tasch E. S. (2006). Treatment of depression for patients with multiple sclerosis in neurology clinics. *Multiple Sclerosis*.

[12] Cetin K., Johnson K., Ehde D., Kuehn C., Amtmann D., Kraft G. (2007). Antidepressant use in multiple sclerosis: epidemiologic study of a large community sample. *Multiple Sclerosis*.

[13] The Centers for Disease Control and Prevention (CDC): National Center for Health Statistics (NCHS). http://www.cdc.gov/nchs/ahcd/index.htm.

[14] The Centers for Disease Control and Prevention (CDC) http://www.cdc.gov/nchs/ahcd/ahcd_estimation_procedures.htm.

[15] The Centers for Disease Control and Prevention (CDC) http://www.cdc.gov/nchs/ahcd/ahcd_data_collection.htm.

[16] Avasarala J. R., O’Donovan CA., Roach S. E., Camacho F., Feldman S. R. (2007). Analysis of NAMCS data for multiple sclerosis, 1998-–2004. *BMC Medicine*.

[17] Zenlea I. S., Milliren C. E., Mednick L., Rhodes E. T. (2014). Depression screening in adolescents in the united states: a national study of ambulatory office-based practice. *Academic Pediatrics*.

[18] Hing E., Schappert S. M., Burt C. W., Shimizu I. M. (2005). Effects of form length and item format on response patterns and estimates of physician office and hospital outpatient department visits. National Center for Health Statistics. *Vital and Health Statistics. Series 2, Data Evaluation and Methods Research*.

[19] List of Antidepressant Medication Classes https://online.statref.com/Document.aspx?.

[20] Anderson J. G., Bartkus D. E. (1973). Choice of medical care: a behavioral model of health and illness behavior. *Journal of Health and Social Behavior*.

[21] Bruce J. M., Hancock L. M., Arnett P., Lynch S. (2014). Treatment adherence in multiple sclerosis: association with emotional status, personality, and cognition. *Journal of Behavioral Medicine*.

[22] Lugaresi A., Rottoli M. R., Patti F. (2014). Fostering adherence to injectable disease-modifying therapies in multiple sclerosis. *Expert Review of Neurotherapeutics*.

[23] Mohr D. C., Boudewyn A. C., Goodkin D. E., Bostrom A., Epstein L. (2001). Comparative outcomes for individual cognitive-behavior therapy, supportive-expressive group psychotherapy, and sertraline for the treatment of depression in multiple sclerosis. *Journal of Consulting and Clinical Psychology*.

[24] Mohr D. C., Likosky W., Bertagnolli A. (2000). Telephone-administered cognitive-behavioral therapy for the treatment of depressive symptoms in multiple sclerosis. *Journal of Consulting and Clinical Psychology*.

[25] Wallin M. T., Wilken J. A., Turner A. P., Williams R. M., Kane R. (2006). Depression and multiple sclerosis: Review of a lethal combination. *The Journal of Rehabilitation Research and Development*.

[26] Mostert J. P., Sijens P. E., Oudkerk M., De Keyser J. (2006). Fluoxetine increases cerebral white matter NAA/Cr ratio in patients with multiple sclerosis. *Neuroscience Letters*.

[27] Mostert J., Heersema T., Mahajan M., Van Der Grond J., Van Buchem M. A., De Keyser J. (2013). The effect of fluoxetine on progression in progressive multiple sclerosis: a double-blind, randomized, placebo-controlled trial. *ISRN Neurology*.

[28] Li I. H., Huang W. S., Shiue C. Y. (2010). Study on the neuroprotective effect of fluoxetine against MDMA-induced neurotoxicity on the serotonin transporter in rat brain using micro-PET. *NeuroImage*.

[29] Solaro C., Bergamaschi R., Rezzani C. (2013). Duloxetine is effective in treating depression in multiple sclerosis patients: an open-label multicenter study. *Clinical Neuropharmacology*.

[30] Mitsonis C. I., Zervas I. M., Potagas C. M. (2010). Effects of escitalopram on stress-related relapses in women with multiple sclerosis: an open-label, randomized, controlled, one-year follow-up study. *European Neuropsychopharmacology*.

[31] Scott T. F., Nussbaum P., McConnell H., Brill P. (1995). Measurement of treatment response to sertraline in depressed multiple sclerosis patients using the Carroll scale. *Neurological Research*.

[32] Ehde D. M., Kraft G. H., Chwastiak L. (2008). Efficacy of paroxetine in treating major depressive disorder in persons with multiple sclerosis. *General Hospital Psychiatry*.

[33] Conner K. O., Copeland V. C., Grote N. K. (2010). Mental health treatment seeking among older adults with depression: the impact of stigma and race. *The American Journal of Geriatric Psychiatry*.

[34] Rost K., Nutting P., Smith J., Coyne J. C., Cooper-Patrick L., Rubenstein L. (2000). The role of competing demands in the treatment provided primary care patients with major depression. *Archives of Familiy Medicine*.

[35] Richardson K., Bennett K., Kenny R. A. (2015). Polypharmacy including falls risk-increasing medications and subsequent falls in community-dwelling middle-aged and older adults. *Age and Ageing*.

[36] Spina E., Santoro V., D'Arrigo C. (2008). Clinically relevant pharmacokinetic drug interactions with second-generation antidepressants: an update. *Clinical Therapeutics*.

